# Practicing for the unexpected: a framework for musical adaptation reserve in expert musicians

**DOI:** 10.3389/fpsyg.2026.1970635

**Published:** 2026-10-27

**Authors:** Rendao Yu, Min Xu

**Affiliations:** 1 Music Department, College of Music and Dance, Jiangxi Institute of Technology, Nanchang, China; 2 Piano Department, School of Music, Jiangxi Normal University, Nanchang, China

**Keywords:** expert performance, motor learning, music performance, musical adaptation reserve, performance preparation, performance simulation, practice variability, representative perturbation practice

## Abstract

Expert musicians devote years to making performance reliable, yet public performance is never a perfect copy of the conditions under which a work was prepared. Instruments differ, rooms alter what performers hear, ensemble partners behave differently, audiences change arousal and attention, and small errors or bodily changes can disrupt a plan that was stable in practice. Performance training therefore faces a problem that is easy to overlook: repeatability under familiar conditions is not the same as robustness under altered conditions. Musical adaptation reserve (MAR) is an organizing framework for the task- and context-specific capacities that allow a performer to preserve pre-specified higher-order musical functions by recalibrating perception, action, attention, expression, and affect when performance conditions depart from those anticipated in preparation. The framework does not assume a single latent adaptive trait. It proposes five partially independent, reciprocally coupled capacities: perceptual calibration, motor flexibility, attentional reallocation, expressive replanning, and affective regulation, supported by structural anchors that stabilize the unfolding performance. To make adaptation testable rather than retrospective, the musical functions to be preserved must be specified before perturbation and evaluated independently of the adjustment used to preserve them. The article further proposes representative perturbation practice (RPP), in which controlled, ecologically relevant variations are introduced after basic stability has been achieved. Eight propositions distinguish practice stability from performance robustness and specify diagnostic tests of solution repertoire, representativeness, and cue redundancy alongside predictions shared with established learning and stress research. No new empirical data are presented. The core endpoint concerns preservational adaptation; artistically valuable transformations require separate prospective criteria or exploratory assessment. The framework tests whether preparation supports conditional responses beyond a preferred solution.

## 1 Introduction

Professional music performance is built on a paradox. Musicians prepare for reliability by repeating, refining, and stabilizing actions, yet they perform in conditions that cannot be made fully stable. A pianist who has prepared on one instrument may encounter a different action, pedal response, register balance, or acoustic relationship at the venue. Ensemble partners may alter timing, projection, or spontaneous response. Even when the physical environment is controlled, the performer is not constant: arousal, fatigue, attention, confidence, and bodily sensation can differ from one performance to the next.

This gap between preparation and performance is not peripheral to expertise. Recent Performance Science studies illustrate several parts of the problem. Shao et al. (2026) used two linked qualitative phases to examine how solo pianists perceive acoustic conditions, interpret them musically, and describe or demonstrate adaptation strategies across contrasting acoustic scenarios. Waddell et al. (2025) developed richer performance simulation around the mismatch between ordinary practice and the visual, aural, social, and psychological conditions of performance. Bae (2026), in an experimental study of 54 piano majors, found that modeling, structural understanding, and narrative imagery produced different patterns of expert-rated expressive change and learner-reported experience; transfer intention was a self-reported outcome, not a demonstration of transfer. Hohagen and Immerz's (2024) systematic review further shows that attentional focus is a consequential but heterogeneous variable in musical learning and performance. Together, these studies motivate a question about portability: how should preparation be evaluated when the conditions supporting a polished performance change?

The usual language of consistency does not fully capture this problem. Consistency is valuable. An expert who cannot reproduce a technically and musically coherent solution has not achieved a basic requirement of performance. Yet consistency can refer to at least two different achievements. Repeatability is the capacity to reproduce a prepared solution under conditions sufficiently similar to those in which it was established. Robustness is the capacity to preserve task-relevant musical functions when important conditions change. A performer can be highly repeatable but fragile: stable in the practice room, yet disrupted by a different acoustic, instrument, audience, or attentional state. Conversely, a performer may allow modest variation in surface parameters while preserving higher-order musical relationships across contexts. In that case, less literal repeatability can coexist with greater functional stability.

This distinction has precedents in motor-learning research. Conditions that make practice performance look successful are not always those that maximize retention or transfer (Schmidt and Bjork, 1992). Variability can support exploration and learning when it is task-relevant, but too much or poorly structured variability can also impair acquisition (Ranganathan and Newell, 2013; Dhawale et al., 2017). The challenge-point framework likewise predicts that the learning value of task difficulty depends on the relation between learner skill and functional task demands (Guadagnoli and Lee, 2004). In sport, representative learning design argues that practice should preserve important information-action couplings of the performance environment rather than rely on decontextualized drills alone (Pinder et al., 2011). None of these ideas transfers automatically to music, because musical performance combines motor precision with open-ended expressive goals. They nevertheless expose a common error: judging preparation only by immediate success in the conditions that produced that success.

The present article develops musical adaptation reserve (MAR), an organizing framework for preserving pre-specified higher-order musical functions when performance conditions change. Reserve names preparedness to recruit alternative functional responses or reorganize a familiar response when its usual action-effect mapping no longer fits. Availability must be demonstrated before a novel perturbation, and effectiveness tested afterward; successful recovery alone does not establish that an option was available in advance. The term therefore denotes conditional readiness, not an accumulated stock, a scalar quantity, or a unitary latent resource analogous to a general cognitive or physiological reserve.

The framework makes three claims. First, stable preparation and adaptive capacity are complementary rather than opposing goals. The case for adaptability is not a case against repetition, automation, or reliable motor organization. Second, adaptability should be trained through representative perturbations: controlled variations that preserve the kinds of informational and action demands likely to matter in real performance. Random novelty is not the target. Third, adaptation should be studied as a profile of interacting but distinguishable capacities rather than presumed to arise from one global factor. The framework comprises five capacities: perceptual calibration, motor flexibility, attentional reallocation, expressive replanning, and affective regulation. Structural anchors, including formal landmarks and performance cues, provide a cross-cutting stabilizing resource.

The article first defines the performance problem and explains why fixed-condition stability can underprepare a musician for altered conditions. It then develops the five MAR components and their interactions, translates them into representative perturbation practice, and states eight testable propositions. The final sections address operationalization, boundary conditions, alternatives, and educational implications. The direct empirical evidence reviewed here is weighted toward piano, especially for acoustic adaptation and expressive practice. Cross-instrument and vocal extensions are therefore hypotheses for future testing rather than established generalizations. The aim is not to claim that every expert performer should practice unpredictably. It is to provide a framework for deciding which forms of stability should be protected, which forms of variability should be introduced, and how those decisions can be tested without defining adaptation after the outcome is known.

## 2 The performance problem: repeatability is not robustness

A polished performance often gives the impression of a fixed plan executed with precision. This impression is partly correct. Expert musicians prepare details, establish tempo relations, coordinate entries, plan memory and breathing where relevant, and refine expressive timing. In an intensive single-pianist case study, Chaffin and Imreh (2002) showed how structural knowledge and deliberately selected performance cues organized preparation and memory. More broadly, Williamon and Valentine (2000) reported relationships between practice characteristics and later performance quality, while Duke et al. (2009) identified distinctive problem-solving behaviors in more effective practicers. These findings do not license a quantitative claim that practice quality matters more than practice time, but they do show that time alone is an incomplete description of preparation. This emphasis is consistent with deliberate-practice research (Ericsson et al., 1993) and with music-specific work on metacognitive planning and self-regulation (Hallam, 2001; Varela et al., 2016).

These findings support deliberate stabilization. An expert performance is not generated from nothing on stage. What needs refinement is the assumption that a stable plan is sufficient because the conditions that support its execution will remain stable. Live performance changes the information available to the performer and the relation between action and effect. Acoustics are an obvious example. A longer reverberation time can alter the perceived clarity of articulation, the timing of pedal release, balance between registers, and the amount of temporal overlap that remains intelligible. Shao et al. (2026) found that pianists did not treat acoustic differences as passive background characteristics. They interpreted them and changed performance decisions. This implies that a performance plan must contain at least some conditional structure: if the room returns sound differently, the performer must know what to listen for and what aspects of action can be modified without disturbing the larger conception.

Instrumental differences create a related problem. A pianist’s intended sound is produced through an action whose mechanical and acoustic consequences vary across instruments. The same familiar movement on two pianos need not yield the same balance, attack, or pedal result. Even when the notation and expressive goal remain identical, the action that realizes them can require recalibration. Whether the same mechanism generalizes to other instruments and voice should be tested with instrument-specific measures rather than assumed from piano examples.

Social and evaluative conditions add another layer. Performance is not only a motor task in a different room. An audition, competition, jury, public recital, or recording session changes the meaning of errors and can alter arousal, self-monitoring, and attentional allocation. The performance-simulation work of Williamon et al. (2014), the musicians’ reports examined by Aufegger et al. (2017), and the next-generation simulator developed by Waddell et al. (2025) all address the fact that ordinary practice does not reproduce many social and psychological demands of performance. Simulation is useful precisely because it can expose a form of specificity: a musician can know the music well while being comparatively unpracticed at performing it under conditions of consequence.

The distinction between repeatability and robustness can be stated in functional terms. Suppose a pianist has developed a phrase with a precise local rubato profile, voicing balance, and pedaling pattern. If the practice instrument and hall remain similar, exact reproduction may work well. In a more resonant room, however, literal reproduction could blur harmonic changes and weaken the perceptual hierarchy. The robust solution is not necessarily the identical one. It is the solution that preserves the higher-order function of the phrase: directional continuity, voice hierarchy, harmonic arrival, and the intended degree of articulation. Surface parameters may change in order for musical relations to remain stable.

This point can be generalized. Surface stability concerns repeatability of observable parameters: exact timing, movement pattern, dynamic profile, breath point, fingering, or pedal duration. Functional stability concerns preservation of what those parameters are serving: intelligibility, structural hierarchy, expressive trajectory, coordination, technical security, or stylistic plausibility. In many performance contexts, expertise requires the second more than the first. The expert’s task is not to vary for its own sake but to know which features are means and which are goals.

Such a distinction is compatible with work on expressive performance. Bae (2026) used the opening of Chopin’s Nocturne in E-flat major, Op. 9, No. 2 with 54 piano majors and found that three practice approaches produced different patterns of change across expert-rated expressive dimensions and learner-reported experience. The study did not test transfer itself; transfer intention was measured by questionnaire. Reybrouck and Schiavio (2024) likewise frame music performance as embodied knowledge acquisition in which sensorimotor and cognitive processes are intertwined. These findings support treating a prepared interpretation as more than one immutable surface realization, while leaving open the stronger question of whether alternative realizations improve robustness under novel constraints.

The concept of robustness also clarifies why variability should not be romanticized. A performance that changes unpredictably because the performer lacks control is not adaptive. Motor-learning research shows that variability can be structured and task-relevant, but it can also reflect noise or interfere with acquisition (Wu et al., 2014; Dhawale et al., 2017; Cardis et al., 2018). For MAR, the important distinction is prospective: before a novel perturbation occurs, can the performer intentionally realize a pre-specified musical function through more than one viable implementation? The lesson is not ‘be less consistent.’ It is ‘be consistent about the right things’.

This leads to a practical diagnostic question. If a performer is asked to play the same work on another instrument, in another acoustic, under evaluative pressure, or after a minor disruption, what must remain stable for the performance still to count as the same artistic conception? The answer will differ by repertoire and performer, but it cannot be decided after the perturbation merely by naming whatever happened to survive. For empirical work, the relevant functions and acceptable tradeoffs must be specified before the altered condition is introduced. MAR begins where the performer can distinguish pre-specified musical functions from the adjustable means that realize them.

## 3 Why stable practice can underprepare performance

Fixed-condition repetition is powerful because it reduces uncertainty. Repetition helps performers identify errors, consolidate movement, refine temporal and dynamic relations, strengthen memory, and free attentional resources for higher-level goals. Any theory of musical adaptation that treats repetition as the problem would therefore be badly specified. The problem arises when repeated success under one set of constraints is taken as evidence that the performer can succeed when those constraints change.

Three learning mechanisms are particularly relevant. The first is specificity. Learning is shaped by the information available during practice. If a pianist always practices with the same auditory feedback, instrument response, seating position, and tempo conception, the resulting control system can become finely tuned to that arrangement. This specificity is not inherently undesirable. Indeed, fine calibration is part of expert performance. Fragility appears when the performer has not learned which changes are consequential and has no practiced response when the familiar mapping is altered.

The second mechanism is guidance by the environment. Practice environments can quietly carry part of the task. Familiar acoustics tell the performer how much sound is returning. A known instrument gives predictable kinesthetic and auditory consequences. A fixed start routine, teacher presence, score position, or room layout can become part of memory retrieval. Chaffin and Imreh's (2002) account of performance cues demonstrates the constructive use of prepared retrieval structures, but the same logic implies that external cues can become dependencies when alternatives are absent. A performer who can restart only from a small number of habitual points or regulate projection only from familiar acoustic feedback may have mastered the prepared situation more than the underlying demand.

The third mechanism is the difference between practice performance and learning. Schmidt and Bjork (1992) argued across learning paradigms that variables which improve performance during acquisition can have different effects on longer-term learning. Guadagnoli and Lee's (2004) challenge-point framework similarly emphasizes that useful learning depends on the informational challenge presented relative to the performer’s skill. When a task becomes too easy or predictable, practice can look fluent while generating little new information. When it becomes too difficult, errors become uninformative because the performer cannot interpret them. The learning problem is therefore to introduce enough challenge to force adaptation without destroying the stable organization on which adaptation depends.

This is where practice variability enters. Ranganathan and Newell (2013) distinguish intervention-induced variability from naturally occurring variability and emphasize that the effects depend on how variability relates to the task. Dhawale et al. (2017) similarly show that the nervous system can use variability as a mechanism of exploration, but that not all variability is useful. The musical application should therefore be conservative: variability should be introduced because a change samples a real performance constraint or exposes an important relation, not because unpredictability is presumed virtuous.

A simple example is acoustic practice. Asking a pianist to play the same phrase once with normal pedaling, once in a deliberately more resonant space, and once while listening from a recording position changes the perceptual problem in a way that maps onto real performance demands. The musician must decide which aspects of the original plan are portable and which need recalibration. By contrast, asking the performer to randomize fingering on every repetition would introduce variability without a clear functional relation to the target performance. The first form can develop a conditional model; the second may simply add noise or prevent consolidation.

Performance simulation provides an intermediate case between repetition and random variability. Williamon et al. (2014) showed how simulated environments can reproduce aspects of recital and audition conditions. Aufegger et al. (2017) reported musicians’ perceptions of simulation training, while Waddell et al. (2025) extended this logic toward richer combinations of acoustic, visual, and social features. Simulation changes practice not because it makes the environment identical to reality, which is impossible, but because it reintroduces information that ordinary practice omits. It gives musicians opportunities to discover how their control changes when consequences become more representative of performance.

The same logic applies to attention. Hohagen and Immerz (2024) found that attentional-focus research in music is still heterogeneous, but the literature is sufficient to reject the idea that attention is a neutral by-product of technical preparation. A performer who can execute only while monitoring one internal cue may become vulnerable when stage demands require attention to shift outward toward ensemble, room, audience, or musical projection. Conversely, a performer trained to attend only to external effects may need internal information when a physical problem emerges. Adaptive control requires flexible allocation rather than loyalty to one attentional location.

Affective conditions also exhibit specificity. Musicians can practice in calm states and then interpret performance arousal as evidence that control is failing. Research outside music shows that the meaning assigned to arousal can influence cognitive and physiological responses. Jamieson et al. (2012) found that reappraising stress arousal as functional produced more adaptive responses in an evaluative setting. Crum et al. (2013) similarly demonstrated that stress mindsets shape responses to stress. These findings do not imply that anxiety is harmless or that severe music performance anxiety should be reframed away. They support a narrower point: preparation that assumes the body will feel the same on stage as in the practice room may leave musicians unprepared for normal fluctuations in arousal.

The consequence is a training asymmetry. Traditional preparation invests heavily in reducing variability in the music and the body, while performance requires the musician to absorb variability in the environment, body, and unfolding event. Expert performers often learn this through experience. They play many halls, instruments, auditions, concerts, and collaborations, gradually building repertoires of adjustment. MAR proposes that some of this learning can be made explicit and deliberately trained rather than left to chance exposure.

## 4 Musical adaptation reserve: the proposed framework

MAR concerns the organization of task-specific adaptive options around a prepared musical performance. Its definition entails four commitments for empirical testing.

First, MAR is an organizing framework, not a claim that all adaptation reflects one latent factor. The five capacities may covary, but they may also dissociate across perturbation types. Second, MAR concerns preservation of pre-specified musical functions, not identical behavior: a performer may change pedaling, movement, attention, local timing, or another implementation variable to protect an agreed function. Third, MAR is bounded and task-specific. Extreme fatigue, injury, equipment failure, impossible task demands, or overwhelming distress may exceed the range in which adaptation is meaningful or safe. Fourth, trainability is a hypothesis. MAR should improve only insofar as repeated, interpretable perturbations build better detection, alternative solutions, and recovery without degrading the stable skill on which adaptation depends.

The five proposed capacities address five functional demands: interpreting changed information (perceptual calibration), varying bodily implementation (motor flexibility), redistributing processing priorities (attentional reallocation), revising expressive relations (expressive replanning), and regulating engagement under altered activation (affective regulation). This selection covers the demands identified in the literature reviewed here; it is a provisional functional decomposition, not evidence that all five are necessary for every perturbation or jointly sufficient for all musical adaptation. A simple instrument change may require mainly perceptual and motor adjustment, whereas an evaluative ensemble disruption may recruit several capacities. Future studies may merge, subdivide, or supplement components if the present distinctions lack discriminant or predictive value. Structural anchors support these processes across the performance and are not a sixth capacity.

The components are partially independent processes whose reciprocal coupling varies with the perturbation, not a fixed hierarchy or serial pipeline. Acoustic classification can guide attention and pedaling, while an attentional shift changes the acoustic information sampled and the resulting action updates calibration. Under evaluation, affective regulation may alter attention before an explicit mismatch is recognized. This architecture permits both anticipatory and feedback-driven adjustment; observed timing and coupling require empirical testing.

Three distinctions are especially important. Perceptual calibration concerns the interpretation and action relevance of information; attentional reallocation concerns which information receives processing priority. A musician can classify excessive reverberation accurately yet fail to shift attention toward harmonic clarity. Motor flexibility concerns bodily means; expressive replanning concerns relations such as phrase direction, balance, or timing priorities. A pianist can change coordination while retaining the same expressive plan, or revise local rubato without demonstrating a different movement repertoire. Affective regulation concerns appraisal and task-effective engagement under activation; attention concerns allocation of processing. Regulating frustration and shifting attention to a partner may co-occur, but neither defines the other.

Operational separation should follow these distinctions: calibration is assessed through discrimination and prospective action prediction; attention through demand-sensitive shifts and their latency or cost; motor flexibility through reliability-distinct implementations; expressive replanning through documented changes in expressive decisions and their relational outcomes; and affective regulation through prospective appraisal and state measures linked to later disruption. Correlations and shared indicators are expected, but the same outcome score should not serve as evidence for every component. Designs can test selective demands and cross-predictor models before claiming independent measurement (Sections 7.2–7.6).

### 4.1 Fixing the musical criterion before perturbation

The phrase ‘higher-order musical functions’ is deliberately operational rather than metaphysical. Before perturbation, a study should translate the relevant musical goal into a small set of observable relational or structural features and fix the scoring dimensions, tolerances, and permitted tradeoffs. In within-person studies, performer-declared goals can define the primary criterion when they are recorded prospectively. In between-group studies, the primary musical-function criterion and tolerance must be common across groups and fixed before group assignment or first exposure; individualized performer goals may be retained as secondary outcomes. Where aesthetic judgment is unavoidable, independent expert raters should apply the same prospectively specified rubric without condition labels.

The prospective criterion defines preservational adaptation: changing the means while retaining a declared musical function. Generative or transformative adaptation instead changes the interpretation or its priorities in response to a new condition. For example, a resonant room might prompt a pianist to replace an initially planned sharply articulated trajectory with a sustained, spacious conception. Such a transformation can be artistically productive even when it fails the original preservation criterion. Failure on that endpoint is therefore not a judgment of artistic failure.

The core MAR endpoint is preservational. Transformative adaptation can enter an extended design only when meta-criteria are fixed before exposure: what may change, which score, stylistic, safety, or ensemble constraints remain binding, what qualifies as a coherent transformation, and how independent listeners will judge it. For example, a study could permit either an articulated or sustained conception while registering structural continuity and harmonic intelligibility as common requirements. The choice and its success would then be assessed under that prospective rule. An unforeseen transformation without such a rule should be reported as an exploratory artistic outcome alongside the unchanged primary score. Criteria can also be revised for a later trial, but not retroactively for the trial that prompted revision.

### 4.2 Perceptual calibration

Perceptual calibration identifies which environmental or bodily differences matter for the musical task and estimates their consequences. Detection often informs reconfiguration, but it need not be a discrete first stage: ongoing attention, action, and affect can alter what is detected, and anticipatory adjustments can precede explicit classification. Calibration is not raw sensitivity. A performer may notice that a hall is reverberant without knowing what should change. It links perceived change to an action-relevant interpretation.

Shao et al. (2026) provide a qualitative musical foundation. Across interviews, observations, and reflective tasks, pianists connected perceived acoustic characteristics with musical understanding and adaptation strategies. The study does not establish how such skill develops causally, but it shows that acoustic differences are not treated merely as passive background characteristics. The theoretical point for MAR is that detection must become action-relevant classification: the performer needs to identify which change matters, what consequence it is likely to have, and which response is worth testing.

Instrument adaptation follows the same structure. A pianist encountering a heavier or differently responsive action must distinguish a mechanical difference from an artistic failure. If the instrument produces a different sound for a familiar sensation, the player must update the relation between felt action and heard result. Comparable claims for other instruments or voice require their own evidence and measurement; they are not inferred here from piano alone.

Calibration can be trained by comparative exposure. Musicians can play the same passage in two rooms or on two instruments while identifying what changed in the resulting sound and which response was effective. Recordings from audience positions can reduce the dominance of the performer’s on-stage perspective. The training goal is not to memorize recipes such as “use less pedal in resonant rooms.” It is to build discriminations: what kind of resonance, in which register, at what tempo, serving which texture, produces which consequence?

A testable prediction follows. A performer with stronger perceptual calibration should not simply report more environmental differences; pre-perturbation classifications should better predict which adjustments will be useful in the changed condition. Perceptual sensitivity without more accurate action prediction would indicate awareness without demonstrated adaptive value.

### 4.3 Motor flexibility

Musical intentions are realized through bodily coordination, but expert movement need not be reduced to one optimal pattern repeated identically. Motor-learning research allows the possibility that more than one coordination pattern can serve a task outcome. Motor flexibility in MAR is the capacity to access and select among viable implementations without assuming that movement diversity is valuable in itself.

Wu et al. (2014) found that task-relevant features of motor variability predicted individual differences in motor learning, Dhawale et al. (2017) reviewed variability as a potential mechanism of exploration, and Ranganathan and Newell (2013) emphasized that intervention-induced variability depends on its relation to the task. At the same time, high variability can impair acquisition (Cardis et al., 2018). MAR therefore predicts neither maximum variability nor a fixed amount of variability. It predicts value in having more than one viable route to a pre-specified function when the task permits it.

For a pianist, this might mean deliberately producing a secure passage through measurably different but safe coordination or timing solutions before a novel instrument is encountered. The point is not to prescribe particular joint patterns from theory. Instrument-specific research must establish which differences are safe, meaningful, and measurable, and whether a broader pre-perturbation solution repertoire predicts smaller transfer costs.

To avoid circularity, MAR does not define an implementation as functional because it later succeeds under perturbation. A pre-perturbation solution repertoire is demonstrated under familiar conditions only when the performer can intentionally realize the same pre-specified musical function through at least two implementations that are distinct beyond measurement and execution unreliability. Distinctness must be defined prospectively from repeated baseline trials: for the chosen implementation measure, the between-implementation difference must exceed a pre-registered reliability-referenced threshold such as the smallest detectable change or the relevant 95% limits of agreement, while both implementations satisfy the same musical-function criterion. Robustness under a later perturbation is then a separate outcome.

Practice can build motor flexibility by varying a narrow constraint after a movement is basically secure and asking which implementation features can change without losing the pre-specified function. The challenge-point framework predicts that such variation must be scaled to expertise (Guadagnoli and Lee, 2004). A learner who has not yet established a basic representation may be destabilized by variations an expert can interpret. Adaptive training should therefore widen the solution repertoire gradually rather than prevent stable coordination from emerging.

### 4.4 Attentional reallocation

Performance demands change where attention is most useful. Attentional reallocation is the capacity to shift the focus and breadth of attention in response to changing task demands without losing continuity. It includes movement between external musical consequences, internal bodily cues, ensemble information, structural landmarks, and evaluative thoughts.

Hohagen and Immerz's (2024) systematic review demonstrates both the relevance and the complexity of attentional focus in musical learning and performance. Findings across music cannot be collapsed into a simple rule that external focus is always superior or that internal attention is inherently disruptive. Musical tasks differ. A performer may need an external sound goal while regulating technique indirectly, yet a sudden physical sensation may require temporary attention to movement. Ensemble playing can demand rapid shifts toward partner cues. Memory uncertainty may make structural landmarks temporarily salient.

The framework therefore treats attentional flexibility, not one preferred attentional target, as the adaptive variable. A musician with low flexibility may become stuck in self-monitoring after a minor problem, or conversely remain absorbed in an expressive image while missing an emerging technical risk. More effective attentional reallocation should appear as an appropriate shift toward task-relevant information followed by return to the broader musical trajectory.

This component is closely related to automaticity. Skilled action benefits when low-level control does not require continuous conscious supervision. But automaticity should not be confused with inaccessibility. Experts need the capacity to intervene when the automated process no longer fits current conditions. The difficulty is to intervene at the right level. Excessive conscious control of well-learned movement under pressure can disrupt performance, while failure to monitor meaningful changes can allow small disturbances to propagate.

Training can make attentional reallocation explicit. A performer can rehearse a passage while alternating between room response, melodic projection, bodily ease, ensemble cue, and formal destination, not to divide attention continuously but to learn what each source of information contributes. Simulation can add evaluative distraction while the performer practices returning attention to task-relevant cues. Short post-performance reflection can identify where attention went during disruption and whether the shift helped.

The predicted signature of MAR is not constant attentional switching. Experts should switch less when the situation is stable because unnecessary reallocation has costs. Greater reserve should appear as faster and more appropriate shifts when conditions demand them, followed by restoration of a task-effective attentional state.

### 4.5 Expressive replanning

Musical performance is not merely a sequence of motor targets. Performers organize phrases, hierarchies, tensions, releases, timbral contrasts, timing relations, and long-range trajectories. Expressive replanning is the capacity to modify local or medium-scale expressive decisions when circumstances change while preserving the larger conception.

This capacity is easy to overlook because interpretation is often prepared as a coherent whole. Once a performer has found a persuasive tempo relation or phrase shape, changing it near a performance can feel like loss of control. Yet some environments make literal preservation self-defeating. A very resonant room may require clearer articulation and different pedal timing. An unusually projecting instrument may permit subtler dynamic effort. An ensemble partner’s spontaneous timing may make a preplanned microtiming profile inappropriate. The adaptive performer distinguishes the artistic principle from its practiced implementation.

Bae's (2026) study is relevant because it demonstrates that expressive practice methods can differentially affect expert-rated performance dimensions and learner-reported cue awareness, personal interpretation, and transfer intention. The final measure is an intention, not evidence of successful transfer, so it cannot carry MAR’s transfer claim. Reybrouck and Schiavio (2024) similarly emphasize the embodied, knowledge-generating character of performance. MAR adds a separate hypothesis: an interpretation is more robust when a performer can reconstruct its pre-specified relational functions under altered constraints rather than only reproduce one practiced surface.

The concept can be operationalized through relational measures fixed before perturbation. If a performer declares a phrase hierarchy and direction toward an identified arrival, altered timing or dynamics can preserve those relations in a new acoustic. Deterioration beyond the registered tolerance counts against preservational adaptation on that endpoint. An artistically persuasive change of conception remains a separate transformative outcome, assessed using the rules in Section 4.1.

This is not a license for arbitrary last-minute change. Replanning remains constrained by the score, style, ensemble commitments, physical feasibility, and the goals fixed for the test. Where multiple outcomes are artistically defensible, the study should compare them against prospective criteria rather than assume a single correct realization. In passages governed by categorical accuracy, adaptation may concern execution rather than interpretation.

Practice for expressive replanning can involve controlled alternatives. Musicians can prepare two viable local solutions, compare professional performances, rehearse the same passage for different room sizes, or deliberately alter one expressive priority while preserving others. The goal is to prevent a single surface realization from becoming synonymous with the musical idea.

### 4.6 Affective regulation

Performance conditions alter affective and physiological state. Affective regulation in MAR is the capacity to maintain or restore task-effective engagement when ordinary fluctuations in arousal, anxiety, excitement, frustration, or bodily activation change. It is not the elimination of arousal, and it is not a claim that severe music performance anxiety can be solved through willpower or reframing. The present framework is restricted to bounded performance demands; clinically significant distress requires appropriate professional support and falls outside a training prescription.

Existing evidence supports a deliberately narrow appraisal claim. Jamieson et al. (2012) showed that reappraising arousal as functional can alter cardiovascular and cognitive responses in evaluative stress, and Crum et al. (2013) showed that stress mindsets can shape stress responses. In music, Rey et al. (2025) found that demand-resource evaluations in classical music students varied with general music performance anxiety, audience presence, and time. These sources do not establish a complete theory of music performance anxiety for MAR; they support only the proposition that how ordinary performance activation is interpreted may contribute to subsequent control.

The framework makes a specific claim about preparation. If musicians practice only when they feel calm and treat any stage arousal as a deviation that must be removed before they can perform, they may create a narrow state-performance mapping. A more adaptable mapping would allow skilled action to coexist with a bounded range of ordinary bodily states. Performance simulation is relevant because it gives musicians experience executing prepared skills while social evaluation, uncertainty, or arousal is present (Williamon et al., 2014; Waddell et al., 2025).

Affective regulation has two functions. The first is interpretive: the performer classifies bodily activation. Increased heart rate may be read as danger, energy, excitement, or a neutral consequence of performance. The second is behavioral: the performer selects a response that protects the task, such as adjusting breathing, attentional focus, pacing, or pre-performance routine. Effective regulation is therefore coupled to the other MAR components. Arousal can change perception, movement, attention, and expressive timing; regulation helps prevent those changes from cascading.

Training should remain bounded and ethical. The framework does not recommend deliberately inducing severe anxiety, sleep deprivation, pain, or unsafe fatigue. Representative perturbation should use tolerable, performance-relevant challenges: mock audiences, recording pressure, limited one-take conditions, realistic audition procedures, or gradual increases in evaluative salience. The aim is familiarity with adaptation, not stress inoculation at any cost.

### 4.7 Structural anchors as a cross-cutting resource

The five capacities require something stable around which adaptation can occur. Structural anchors provide that stability. They include formal landmarks, harmonic destinations, ensemble cues, memorized start points, auditory images, and other performance cues that allow the musician to locate the current event within the larger structure.

Chaffin and Imreh (2002) provide an intensive single-pianist case study of expert memory while learning the third movement (Presto) of J. S. Bach’s Italian Concerto. Their study showed how performance cues can organize preparation and memory; it did not test MAR. A MAR extension would map several cues before perturbation and then test whether recovery remains possible when one cue source is weakened. In that design, a structural anchor is not whatever cue happens to rescue a performance after the event; its availability is documented beforehand.

Redundancy is therefore a specific hypothesis rather than an established result from the Chaffin and Imreh case. A performer who prospectively identifies multiple independent anchors may have alternative routes for maintaining continuity if one becomes unreliable. Proposition 8 tests whether such pre-mapped cue redundancy predicts recovery beyond baseline memory quality.

Figure 1 depicts partially independent, reciprocally coupled capacities rather than a fixed sequence or hierarchy. Their coupling varies with perturbation type; perceptual classification may guide a motor or expressive response, while action, attentional weighting, and affect update subsequent classification. Structural anchors support the network throughout. The seven perturbation categories match Section 5.1 and Table 1. Outcomes are evaluated against the applicable criterion fixed before exposure, including separate criteria for prospectively assigned goal conditions, and inform subsequent recalibration.

**Figure 1:**
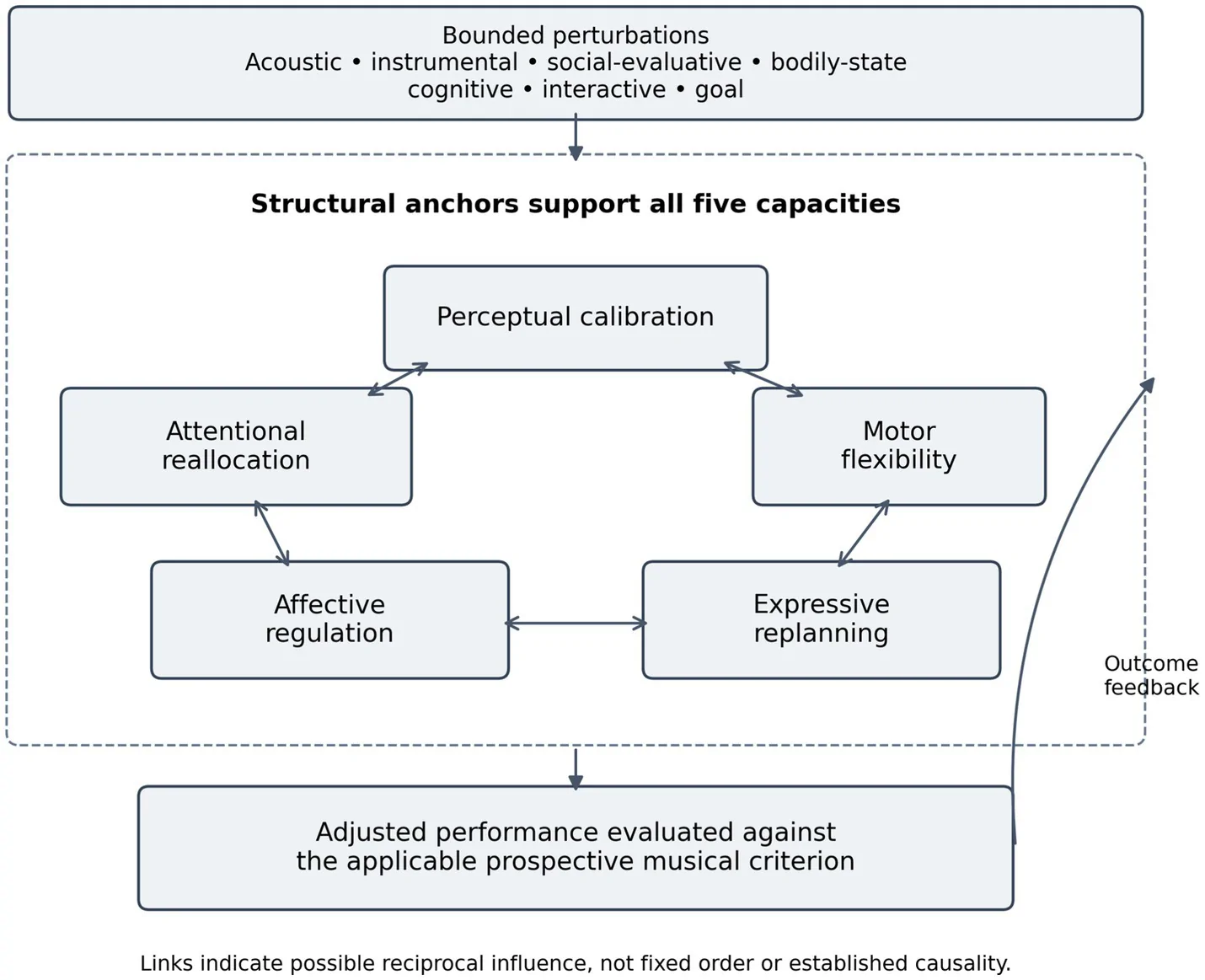
Musical adaptation reserve as a network of partially independent, reciprocally coupled capacities under bounded performance perturbation. Acoustic, instrumental, social-evaluative, bodily-state, cognitive, interactive, and goal perturbations can engage different component profiles. Bidirectional links indicate possible reciprocal influence, not established causal paths, equal coupling, or a mandatory sequence. Structural anchors support all five capacities across the performance and are not a sixth capacity or a downstream stage. Adjusted performance is evaluated against the applicable musical-function criterion fixed before exposure; outcomes inform subsequent recalibration across the network. Goal conditions each have their own prospective criterion. Transformative outcomes are evaluated separately as specified in Section 4.1.

**Table 1:** Representative perturbations and their proposed adaptive targets.

Perturbation category	Representative change	Primary MAR capacities	Structural-anchor/criterion role
Acoustic	Contrasting rooms, audience perspectives, or acoustic simulation	Perceptual calibration; expressive replanning	Pre-specify the musical function and use structural anchors to maintain phrase or formal direction.
Instrumental	Different piano action or pedal response; other instruments require validated, safe analogues	Motor flexibility; perceptual calibration	Separate the intended function from one familiar action-effect mapping.
Social-evaluative	Mock audition, one-take recording, small audience, or jury simulation	Affective regulation; attentional reallocation	Apply the same prospective musical criterion independently of reported arousal.
Bodily-state	Bounded, nonclinical variation in ordinary performance-relevant activation or bodily state	Affective regulation; attentional reallocation	Exclude unsafe fatigue, pain, sleep deprivation, and clinically significant distress; retain the same prospective musical criterion.
Cognitive	Unexpected start point, benign interruption, or controlled reduction of cue availability	Attentional reallocation; perceptual calibration	Test prospectively mapped cue redundancy; structural anchors remain a cross-cutting resource.
Interactive	Musically plausible changes in a partner’s timing, balance, or emphasis	Perceptual calibration; attentional reallocation; expressive replanning	Pre-identify ensemble or formal cues relevant to coordination.
Goal	Different pre-registered performance priorities for the same material	Expressive replanning; perceptual calibration	Fix a separate musical-function criterion for each goal condition before exposure.

### 4.8 Relation to resilience, flexibility, expertise, and self-regulation

MAR is an integrative framework with additional operational and testable commitments, rather than a claim to an independent faculty or an exclusively owned prediction. Combining adaptive expertise, representative learning design, motor flexibility, self-regulation, and performance-cue accounts can explain much of the proposed behavior. MAR’s incremental contribution is to organize those processes around a common unit of analysis: an unfolding musical performance under a specified perturbation, scored against a musical function declared beforehand and independently of the implementation used. It separates available responses from later robustness and baseline quality from perturbation cost, recalibration, and transfer. The diagnostic tests ask whether reliability-distinct solution repertoire, independently classified representativeness, and prospectively mapped cue redundancy predict those outcomes beyond relevant alternatives. These commitments are attributable to MAR’s present formulation but are compatible with neighboring theories; confirmation would support their usefulness, not establish theoretical exclusivity.

Table 2 compares this contribution with neighboring constructs. Adaptive expertise addresses effective and innovative responses to novel situations across a domain (Bohle Carbonell et al., 2014); adaptive performance provides a multidimensional taxonomy of adjustment to changing workplace demands (Pulakos et al., 2000). MAR narrows their unit of analysis to a specified musical perturbation and a prospective musical criterion. It does not claim that either broader account excludes musical adaptation.

**Table 2:** MAR in relation to neighboring accounts and the limits of its incremental contribution.

Neighboring account	Shared explanatory content	MAR contribution and diagnostic test
Adaptive expertise (Bohle Carbonell et al., 2014)	Effective responses to novelty; possible innovation.	Specified musical perturbation and prospective function; test incremental component prediction, not domain-general innovation.
Adaptive performance (Pulakos et al., 2000)	Multidimensional adjustment to changed demands.	Independent musical-function endpoint separates adjustment behavior from functional success.
Motor abundance and degeneracy (Latash, 2012; Seifert et al., 2016)	Multiple configurations can support one function.	Reliability-distinct, intentionally available implementations tested before novelty; Proposition 2 tests later robustness beyond repeatability.
Representative learning design (Pinder et al., 2011)	Practice preserves target information-action relations.	Independent ex-ante classification and prospective musical transfer criterion; Proposition 3 compares matched variability.
Self-regulation (Hallam, 2001; Varela et al., 2016)	Planning, monitoring, evaluation, and adjustment.	Relates prepared options and online process measures to perturbation cost, recalibration, and transfer.
Performance cues (Chaffin and Imreh, 2002)	Prepared retrieval structures organize memory.	Cue availability documented before degradation; Proposition 8 tests recovery beyond baseline memory skill.
Resilience	Functioning or recovery through adversity over broader timescales.	Bounded online musical response; affective engagement measured separately from function preservation.
Across accounts	Many predictions are compatible or shared.	No prediction is claimed as exclusive; added value depends on reliable improvement over corresponding established measures.

Motor abundance and degeneracy explain how multiple degrees of freedom or structurally different configurations can support the same function (Latash, 2012; Seifert et al., 2016). MAR adds the proposed test that intentionally available implementations, demonstrably distinct beyond unreliability before perturbation, predict later robustness. Representative learning design supplies RPP’s information-action constraint (Pinder et al., 2011); MAR specifies its ex-ante classification and music-specific transfer endpoint. Self-regulation and performance cues support preparation and monitoring (Hallam, 2001; Varela et al., 2016; Chaffin and Imreh, 2002), while resilience addresses functioning through adversity over broader timescales. None is treated as an inadequate account merely because its vocabulary differs.

The framework earns explanatory value only if its prospective measures improve prediction beyond baseline skill, relevant experience, confidence, anxiety, and the corresponding measures supplied by neighboring accounts. Propositions 2, 3, and 8 provide diagnostic tests, not predictions unique to MAR. If established measures explain the same outcomes equally well and the MAR operationalization adds no reliable information, its role should remain descriptive and integrative rather than a distinct explanation.

## 5 Representative perturbation practice

If MAR is trainable, practice must occasionally contain the need to adapt. Representative perturbation practice (RPP) refers to training in which controlled variations are introduced after basic task stability has been established, with the goal of strengthening detection, diagnosis, and reconfiguration. RPP adopts representative learning design’s requirement that practice preserve task-relevant information-action relations (Pinder et al., 2011) but does not equate representativeness with realism or difficulty.

Representativeness must be tested ex ante. Before outcome data are collected, the investigator must specify (1) the target performance context, (2) the constraint or information variable changed by the perturbation, (3) the action adjustment that this information can plausibly guide, and (4) why the practice manipulation preserves the same information-action relation expected in the target context. Independent domain experts should classify the mapping without outcome information, using a pre-registered agreement criterion; a manipulation that fails that criterion is not analyzed as representative. This operational test defines the RPP condition in Proposition 1 and the representative condition in Proposition 3.

### 5.1 A taxonomy of representative perturbations

Seven perturbation categories are used consistently in the framework.

Acoustic perturbations alter reverberation, clarity, monitoring, or apparent distance. They primarily engage perceptual calibration and expressive replanning.

Instrumental perturbations alter the action-effect mapping, such as differences in piano action or pedal response where safe and feasible. They primarily engage motor flexibility and perceptual calibration.

Social-evaluative perturbations alter observation, consequence, or evaluative salience through conditions such as a mock audition, one-take recording, small audience, or jury simulation. They primarily engage affective regulation and attentional reallocation.

Bodily-state perturbations concern bounded, nonclinical changes in ordinary performance-relevant activation or bodily state. They exclude pain, dangerous fatigue, sleep deprivation, and deliberate induction of clinically significant distress, and primarily engage affective regulation and attentional reallocation.

Cognitive perturbations alter cue availability or recovery demands through conditions such as an unexpected start point, benign interruption, or controlled reduction of a normally available cue. They primarily engage attentional reallocation and perceptual calibration while directly testing the use of structural anchors.

Interactive perturbations arise from musically plausible changes introduced by another performer, including timing, balance, or expressive emphasis. They primarily engage perceptual calibration, attentional reallocation, and expressive replanning.

Goal perturbations assign different intended musical functions to the same material, with a separate criterion fixed for each condition before exposure. They primarily engage expressive replanning and perceptual calibration. They test adaptation to a prospectively assigned goal; they do not license replacing a failed primary criterion after an unanticipated interpretive transformation.

Table 1 uses these same seven categories and treats structural anchors as a cross-cutting resource rather than an additional MAR component.

### 5.2 Stabilize before perturbing

The most important limitation on RPP is sequencing. Variability has learning value only when the performer has enough control to interpret it. Guadagnoli and Lee's (2004) challenge-point framework predicts that functional difficulty should be matched to skill, while evidence on motor variability warns that excessive variation can interfere with learning (Cardis et al., 2018). RPP therefore begins with stabilization.

Stabilize means establishing a workable representation of the music, basic technical security, and a prospective record of the musical functions to be preserved. It does not mean perfecting every local parameter before adaptation begins. Once a performer can distinguish the intended function from ordinary baseline variability, a narrow perturbation can be introduced.

The next step is perturb: change one relevant condition while keeping others relatively stable. A pianist might change room acoustics while keeping instrument and social context constant. Isolating one perturbation initially makes diagnosis easier and reduces the temptation to invent a *post hoc* explanation for a complex failure.

The performer then diagnoses what changed: what is different in the result, why the previous solution no longer fits, and which MAR component is implicated. Diagnosis is crucial because exposure alone can produce superstition. A performer may attribute difficulty to the wrong feature and develop an unnecessary ritual.

Next, comes reconfigure: alter movement, attention, expression, or regulation strategy while attempting to preserve the functions fixed before perturbation. The smallest effective change is a sensible default because it protects established skill from needless reorganization, but its success must still be judged against the prospective criterion.

Finally, the performer re-tests and transfers. A successful adjustment is tested again and then compared across another condition. The training question is whether the performer has learned a conditional relation, not merely a new fixed solution for the second environment.

This yields a practical sequence: stabilize → specify → perturb → diagnose → reconfigure → re-test → transfer. The sequence is not a rigid protocol; ‘specify’ is included to prevent the criterion for successful adaptation from being chosen after the outcome.

### 5.3 From one perturbation to combined simulation

Real performance rarely changes one variable at a time. A competition may combine unfamiliar instrument, dry acoustic, panel of judges, time pressure, and elevated arousal. If RPP never combines perturbations, it risks becoming another artificial training environment. Yet introducing maximal complexity immediately can make failure uninterpretable.

A graded simulation approach solves this problem. Musicians can begin with isolated perturbations, then combine two, then move toward full representative simulations. Waddell et al. (2025) argue for next-generation simulation precisely because realistic performance contains interacting visual, aural, and social dimensions. MAR predicts that combined simulations are most useful after performers have learned some component-level responses. The aim is not only to withstand pressure, but to coordinate several adaptation processes simultaneously.

For example, a pianist preparing an audition might first rehearse across two instruments, then add one-take recording, then add a small panel, and finally perform in an unfamiliar hall under realistic timing rules. After each stage, the musician identifies which adjustments were effective and which demands competed for attention. This produces a map of vulnerability rather than a binary verdict of “ready” or “not ready”.

### 5.4 The role of reflection and external feedback

MAR may include tacit processes, but concise reflection can connect perturbation to consequence. A useful record asks: What changed? Which pre-specified function was affected? What adjustment was attempted? What stayed stable? What evidence suggests the adjustment helped? What should be tested next? This is compatible with music-specific self-regulation research, including microanalytic reflection and pedagogical tools for self-monitoring (López-Íñiguez and McPherson, 2024; Utermohl de Queiroz et al., 2025). RPP adds a narrower requirement: reflection is tied to a controlled perturbation and a prospective success criterion.

External feedback can improve calibration because performers do not always have access to the audience perspective. Recordings, teacher observations, pre-specified acoustic measures, or blinded expert judgments can reveal mismatches between felt and heard effects. The role of feedback is not to supply a universal solution but to test the performer’s diagnosis and improve the mapping between changed conditions and musical consequences.

## 6 Testable propositions and research designs

The framework yields eight propositions, summarized in Table 3. Propositions 1 and 4 are primarily compatibility tests with established acquisition-transfer and challenge-point predictions; Propositions 5, 6, and 7 extend simulation, appraisal, and acoustic-adaptation work. Propositions 2, 3, and 8 are diagnostic of MAR’s proposed operationalization through solution repertoire, ex-ante representativeness, and mapped cue redundancy; they are not theoretically exclusive. Between-group tests use the common prospective primary criterion and tolerance defined in Section 4.1.

**Table 3:** Testable propositions derived from the musical adaptation reserve framework.

Proposition	Core prediction	Primary comparison	Result that counts against the claim
Proposition 1: Acquisition-transfer tradeoff	Fixed practice should look more stable at acquisition; ex-ante representative RPP should show smaller transfer cost.	Fixed-condition practice vs. RPP passing the Section 5 representativeness test, using the same prospective musical criterion.	No transfer advantage for RPP under matched exposure.
Proposition 2: Reliability-distinct solution repertoire	A pre-perturbation repertoire whose implementations differ beyond a reliability-referenced threshold should predict robustness beyond surface repeatability.	Reliability-distinct repertoire and repeatability entered together with baseline skill and relevant experience.	Repertoire adds no predictive value, or repeatability predicts transfer equally well or better across replications.
Proposition 3: Ex-ante representative perturbation advantage	Perturbations classified as representative before outcome testing should outperform matched nonrepresentative variability on target transfer.	Representative vs. nonrepresentative perturbations matched for exposure, variability, and nominal difficulty.	Equivalent transfer across the matched conditions.
Proposition 4: Expertise-calibrated perturbation range	Useful perturbation range should widen with expertise/task familiarity.	Skill level × perturbation range on delayed transfer.	No predicted interaction, or wider variability benefits less-established skills equally.
Proposition 5: Staged simulation	Isolated-to-combined simulation should transfer better than no simulation or immediate maximal complexity.	No simulation vs. full simulation vs. staged simulation.	No staged advantage under equivalent exposure.
Proposition 6: Arousal appraisal and disruption	Within nonclinical stress, compatible appraisal should predict less disruption and better preservation of registered functions.	Prospective appraisal, attention, and robustness measured separately.	Appraisal does not predict the proposed pathway or robustness once relevant covariates are controlled.
Proposition 7: Acoustic adaptation training	Interpretable acoustic variation plus diagnosis should reduce transfer cost to an unfamiliar room.	Single-acoustic practice vs. varied-acoustic diagnostic practice using the same prospective musical criterion.	No smaller transfer cost for varied diagnostic practice under matched time and exposure.
Proposition 8: Prospectively mapped cue redundancy	More prospectively mapped independent anchors should predict faster or cleaner recovery when one cue source is weakened.	Pre-mapped cue diversity vs. recovery under controlled cue degradation.	Cue diversity does not predict recovery beyond baseline memory skill.

### 6.1 Proposition 1: acquisition-transfer tradeoff

Fixed-condition repetition should maximize local practice stability more than RPP, whereas RPP should yield greater robustness under novel but related conditions. This dissociation is expected from established learning-transfer work and is not unique evidence for MAR (Schmidt and Bjork, 1992). The RPP manipulation must satisfy the ex-ante representativeness test in Section 5 before outcome analysis. In between-group designs, both groups must be scored against the same primary musical-function criterion and tolerance fixed before assignment or first exposure.

### 6.2 Proposition 2: reliability-distinct solution repertoire

A pre-perturbation repertoire of reliability-distinct viable implementations should predict robustness better than surface repeatability. Before transfer, repeated familiar-condition trials establish the unreliability of the chosen implementation measure. Two implementations count as distinct only when their difference exceeds the pre-registered reliability-referenced threshold defined in Section 4.3, while both satisfy the same pre-specified musical-function criterion. The outcome is robustness under a later unfamiliar perturbation.

Solution-repertoire and surface-repeatability measures should enter the same predictive model alongside baseline skill and relevant experience. If reliability-distinct repertoire adds no predictive value, or surface repeatability predicts transfer equally well or better across replications, Proposition 2 is not supported.

### 6.3 Proposition 3: ex-ante representative perturbation advantage

Perturbations that pass the ex-ante representativeness test in Section 5 should enhance target transfer more than nonrepresentative perturbations matched for nominal difficulty, exposure, and amount of variability. This prediction applies representative learning design to music while making the representative classification independent of later performance outcomes (Pinder et al., 2011).

A controlled comparison should match total practice, exposure count, and difficulty as closely as possible and use the same prospective musical-function criterion across groups. If the representative and matched nonrepresentative groups show equivalent transfer, the proposed representativeness advantage is not supported.

### 6.4 Proposition 4: expertise-calibrated perturbation range

The optimal perturbation range should increase with expertise and task familiarity. Learners with an unstable representation need more consistent conditions; experts can interpret a broader range because they possess richer criteria and more practiced solutions. This is principally a challenge-point prediction that constrains MAR rather than uniquely supporting it (Guadagnoli and Lee, 2004).

A developmental design could compare intermediate, advanced, and professional musicians across low, moderate, and high representative perturbation ranges, with delayed transfer as the primary outcome. The predicted expertise-by-variability interaction should be specified in advance. If wider perturbation is equally beneficial at all skill levels, the proposed sequencing constraint would need revision.

### 6.5 Proposition 5: staged simulation

Staged simulation should produce more transferable adaptation than either no simulation or immediate maximal-complexity simulation when the final task contains interacting demands. If simulation is too simple, it may omit relevant information; if it is maximally complex from the outset, performers may be unable to identify which change caused difficulty. This proposition extends existing simulation research rather than uniquely identifying MAR (Williamon et al., 2014; Aufegger et al., 2017; Waddell et al., 2025).

In audition preparation, one group could practice normally, a second move directly into a full simulation, and a third progress through isolated then combined representative constraints. Pre-registered outcomes should include final transfer cost and diagnostic accuracy. No staged advantage under equivalent exposure would count against this sequencing claim.

### 6.6 Proposition 6: arousal appraisal and disruption

Within bounded, nonclinical performance stress, appraisals that treat ordinary activation as compatible with effective performance should be associated with less attentional disruption and better maintenance of pre-specified functions than appraisals that treat the same activation as evidence of impending failure. This prediction builds on reappraisal and stress-mindset research (Jamieson et al., 2012; Crum et al., 2013) and should be tested alongside music-specific demand-resource evaluations (Rey et al., 2025).

This proposition does not predict that reappraisal eliminates severe music performance anxiety, and MAR is not a treatment model. Studies should report anxiety severity and avoid exposing participants to unsafe levels of stress. Within ordinary evaluative conditions, mediation can be tested only if appraisal, attentional disruption, and performance robustness are measured separately; failure of the proposed pathway should narrow the affective component.

### 6.7 Proposition 7: acoustic adaptation training

Musicians trained across interpretable acoustic conditions should show smaller disruption in an unfamiliar acoustic than musicians trained in one acoustic, provided total practice is matched. Shao et al. (2026) offer a qualitative foundation by documenting how pianists perceive, interpret, and describe adaptation to contrasting acoustic scenarios; they do not provide causal evidence that training develops the capacity. MAR adds that training prediction.

A field or simulation study could use multiple contrasting spaces, with outcomes fixed before testing: expert ratings, relevant audio measures, pedal or timing relations where appropriate, self-reported diagnostic accuracy, and time to reach the pre-specified criterion. The key outcome is transfer cost to a new room. Simple exposure should be distinguished from training that requires diagnosis and re-test.

### 6.8 Proposition 8: prospectively mapped cue redundancy

Performers with more prospectively mapped, independently identifiable structural or perceptual anchors should recover more effectively when one cue source is weakened. Chaffin and Imreh (2002) established the importance of performance cues in one expert pianist; MAR makes the separate, testable prediction that cue redundancy documented before perturbation predicts recovery under controlled cue degradation.

Researchers can create cue maps before testing and then weaken one cue source through an unexpected start point, reduced visual information, altered feedback, or a benign interruption. Recovery time, continuity, error propagation, and route selection can then be assessed. If pre-mapped cue diversity does not predict recovery beyond baseline memory skill, Proposition 8 is not supported.

## 7 Operationalizing musical adaptation reserve

Measurement must distinguish adaptation from baseline quality. MAR assessment separates familiar-condition level, first-exposure perturbation cost, recalibration, and novel transfer; a single performance score cannot establish which process accounts for success.

### 7.1 Core measurement sequence

The core measurement sequence contains four elements. First is a stable familiar-condition baseline based on repeated observations. Second is perturbation cost: the change on first exposure to a relevant altered condition, before substantial recalibration can occur. Third is recalibration gain: the amount and speed of recovery after the performer has information about the changed condition and an opportunity to adjust. Fourth is transfer robustness: performance in a new condition not directly trained. Each outcome should be defined against the musical functions and tolerances specified before perturbation.

A small initial perturbation cost is not the only adaptive pattern: a performer may incur substantial disruption but recover rapidly. Studies should pre-register meaningful deterioration and recovery, using repeated baseline variability to inform thresholds. Component predictors and adjustment behavior must be measured separately from the musical endpoint.

### 7.2 Measuring perceptual calibration

Perceptual calibration can be assessed through discrimination, classification, and action prediction. Musicians can compare rooms, instruments, or monitoring conditions and estimate how those differences should affect performance. Accuracy can be evaluated against pre-specified physical measures where available, but the central MAR criterion is prospective: does the judgment predict the direction or type of adjustment that will be useful? An acoustic study might ask a pianist to estimate relative clarity or decay, state the expected consequence for an agreed musical function, and then test that prediction.

Self-report alone is insufficient. Experts can possess tacit calibration they cannot describe fluently, while confident verbal reports may be wrong. Studies should therefore triangulate interview or rating data with acoustic outcomes and behavior. Shao et al. (2026) provide a qualitative foundation for this approach by showing how pianists connect perceived acoustic characteristics to decisions. Future research can add repeated-measures designs to test the accuracy and speed of those mappings.

### 7.3 Measuring motor flexibility

Motor flexibility should be measured as the capacity to reorganize coordination while maintaining a task-relevant result. Motion capture, wearable sensors, instrument-interface data, or video analysis can quantify changes in movement. The interpretation must remain outcome-relative. Greater kinematic variability is not automatically evidence of reserve.

Analysis compares implementation changes with the registered musical outcome. Intentionally different coordination patterns that each satisfy the same criterion demonstrate flexibility; instability of both implementation and outcome does not. The reliability threshold in Section 4.3 distinguishes implementation differences from measurement or execution noise (Wu et al., 2014; Dhawale et al., 2017).

The pre-perturbation repertoire test in Section 4.3 establishes availability under familiar conditions; a later unfamiliar trial independently establishes whether that availability predicts robustness.

### 7.4 Measuring attentional reallocation

Attention is difficult to infer from performance alone. A multimethod approach is preferable. Thought probes immediately after short trials, eye tracking where ecologically feasible, secondary-task costs, retrospective reports, and experimentally manipulated focus instructions can all provide partial evidence. The key MAR variable is not an average internal-versus-external focus score. It is the appropriateness and speed of attentional change when task demands shift.

For example, an ensemble perturbation might make a partner’s timing more informative, while a physical perturbation might briefly make proprioceptive information more useful. A performer with greater attentional reserve should increase weighting of the relevant source and then return to a broader performance focus once the problem is stabilized. Hohagen and Immerz (2024) show why measurement choices matter in musical attentional-focus research; MAR adds a within-person, demand-sensitive prediction.

### 7.5 Measuring expressive replanning

Expressive measures should apply the prospective relational rubric defined in Section 4.1. Where several interpretations are defensible, preservational and transformative outcomes should be reported separately rather than collapsed into one correctness score.

Audio features can quantify timing, dynamics, articulation, balance, spectral properties, and pedaling where available. Expert listeners can judge pre-specified dimensions such as structural clarity, expressive coherence, stylistic plausibility, or continuity. The strongest evidence of expressive replanning occurs when implementation changes, the prospective function remains within criterion, and the change is linked to the altered constraint rather than to a retrospective redefinition of the goal.

Named repertoire can anchor new designs without implying that existing studies tested MAR. Bae's (2026) Chopin excerpt could support a prospective phrase-relation test under acoustic change; Chaffin and Imreh's (2002) Bach example could support a pre-mapped cue-degradation study. Each extension requires its own criteria and independent evidence.

### 7.6 Measuring affective regulation

Affective regulation can be assessed through state anxiety, perceived demands and resources, subjective arousal, heart rate and heart-rate variability, electrodermal measures, and behavioral continuity. No single physiological pattern should be treated as a universal marker of effective regulation. The important question is whether changes in affective state predict disruption and whether the performer can maintain task-relevant control despite bounded increases in arousal.

Simulation provides a useful research platform because evaluative intensity can be manipulated while repertoire and many physical conditions remain stable. Studies can compare ordinary practice, low-stakes performance, and progressively more representative evaluation. MAR predicts that musicians with greater affective reserve will not necessarily report less arousal. They should show a weaker coupling between arousal change and loss of attentional, motor, or expressive control.

The appraisal prediction should likewise avoid turning an outcome into its own explanation. If two performers experience similar activation but differ in appraisal, the proposed pathway is supported only if appraisal measured before or during the perturbation predicts later attentional disruption or robustness. Retrospective statements made after performance should not substitute for the predictor.

### 7.7 Structural anchors and recovery dynamics

Structural anchoring can be measured through restart tasks, memory reports, performance-cue mapping, and controlled interruptions. Researchers can ask musicians to identify reliable entry points, perform from unexpected locations, or continue after a benign disruption. The dependent variables can include time to reorientation, number of propagated errors, continuity of pulse and phrase, and the variety of cues used to recover.

A particularly informative design would compare cue maps created during practice with behavior under perturbation. If performers who prospectively encode multiple formal, harmonic, auditory, or motor anchors recover more effectively when one cue source is degraded, this would support the redundancy prediction. Chaffin and Imreh (2002) provide a single-pianist basis for studying prepared performance cues; the claim that cue diversity improves perturbed recovery remains a MAR hypothesis requiring broader evidence.

### 7.8 Component profiles, strategy choice, and falsification

Report perturbation-specific component measures and robustness separately. The five capacities need not share a latent dimension, and strength in one perturbation class need not generalize to another.

Latent-variable models may test shared covariance, but discriminant and incremental validity take priority: do the process distinctions in Section 4 identify different predictors, and does their coupling add information beyond established measures? A common factor should not be assumed in constructing scores.

Capacity and strategy choice should also be distinguished. A decision to revise artistic priorities can be recorded as a transformative or secondary outcome under Section 4.1; it cannot retrospectively alter the preservational endpoint.

The strongest tests of MAR will combine controlled perturbations, repeated observations, multimodal measurement, and prospectively fixed musical functions. The framework should be narrowed if component measures fail to predict perturbation cost, recalibration, or transfer beyond baseline skill, relevant experience, confidence, and anxiety. More specifically, repeated null or reversed results for Propositions 2, 3, and 8 under adequately powered, representative designs would remove much of the framework’s distinctive empirical content.

## 8 Boundary conditions, risks, and alternative explanations

MAR is intended to narrow, not broaden indiscriminately, the case for variability. Several boundary conditions are essential.

### 8.1 Stability remains a prerequisite

A musician cannot adapt a skill that has not been sufficiently acquired. Early technical learning, unfamiliar notation, and newly formed motor coordinations may require stable practice conditions. Excessive perturbation can prevent the learner from discovering what a successful action feels and sounds like. Cardis et al. (2018) provide a general motor-learning warning that high variability can impair learning. RPP should therefore be introduced after the performer has a workable baseline, and the threshold will differ across components of the same piece.

### 8.2 Not every performance variable should be flexible

Some constraints are categorical. Pitch accuracy, ensemble entries, safe physical technique, score requirements, and coordination commitments may tolerate little variation. Others are relational and adaptable. MAR requires explicit classification of what can change. A framework that celebrates flexibility without identifying invariants would undermine the reliability it is meant to protect.

### 8.3 Adaptation should not become hypervigilance

Training performers to monitor every environmental change could increase self-consciousness and reduce automaticity. MAR therefore predicts selective sensitivity, not continuous surveillance. Zielke et al. (2023) showed that flow during music performance can be entered and disrupted in relation to identifiable musical features and attentional demands; their study does not test RPP, but it reinforces the need to treat focused performance state as something deliberate disruption can disturb. RPP studies should therefore measure monitoring cost, flow or absorption where relevant, and whether performers learn to ignore nonconsequential variation rather than simply notice more.

### 8.4 Simulation can create false confidence

A simulation is a model of performance, not performance itself. If musicians become comfortable in one simulated setup, they may simply create another fixed-condition skill. Waddell et al. (2025) explicitly pursue richer simulation because performance environments are multidimensional. MAR adds that simulation should vary and should include transfer beyond the simulator. The test of simulation is not comfort within it but adaptation outside it.

### 8.5 Individual differences will be substantial

Arousal tolerance, sensory sensitivity, injury history, instrument familiarity, and prior performance experience may shape which perturbations are useful. Some performers may adapt readily in one domain and poorly in another. MAR should therefore be represented as a component profile rather than a single global score. This is a design commitment, not a temporary inconvenience awaiting a latent factor.

### 8.6 Adaptation may arise from accumulated experience rather than special training

One alternative explanation is that experts develop many of the capacities grouped by MAR naturally through accumulated performance experience. This is plausible, and the framework does not require deliberate RPP to be the only route. It predicts that structured perturbation can make such learning more explicit or efficient, a claim that requires longitudinal comparison with ordinary exposure rather than assertion.

### 8.7 The apparent benefits of RPP may reflect confidence rather than adaptive control

Simulation and varied practice may make musicians feel more prepared. Greater confidence could itself improve performance. This is not inconsistent with MAR, but it is a competing mechanism if performance gains occur without better detection or reconfiguration. Studies should therefore measure both confidence and component-level adaptation. MAR predicts that robustness will be partly explained by observable recalibration processes, not only expectancy.

### 8.8 Ecological validity and experimental control will conflict

The more representative a performance environment becomes, the harder it is to isolate causal variables. Acoustic, social, motor, and affective factors interact. Research should therefore combine controlled component studies with high-fidelity simulations and field studies. The framework is best evaluated through converging evidence rather than one decisive experiment.

### 8.9 Ethical and health limits are non-negotiable

Performance education has a history of normalizing excessive workload, pain, and psychological pressure. MAR must not become another rationale for exposing musicians to harmful conditions. Perturbations should be bounded, reversible, and appropriate to the learner. Pain, clinically significant anxiety, dangerous fatigue, or injury risk should trigger support and modification, not “adaptation training.” Performance health is a core part of performance science, and robustness should never be defined as the ability to tolerate unsafe practice.

## 9 Implications for performance education

MAR shifts the educational question from ‘Can the student play this reliably?’ to ‘What happens when the conditions that supported reliability change?’ The first question remains necessary. The second becomes useful only after the student has enough stability for a perturbation to be interpretable.

For teachers, the framework suggests small transfer probes rather than constant unpredictability. After a passage is stable and its musical functions have been stated, one representative condition can be changed and the student asked to diagnose the consequence before attempting a new solution. This retains the reflective strengths of self-regulated music practice while tying reflection to an explicit transfer test (López-Íñiguez and McPherson, 2024; Utermohl de Queiroz et al., 2025).

The framework also changes how disturbances are interpreted in training. A problem during perturbation practice is not automatically evidence of poor preparation; it can reveal a context-bound dependency that fixed practice concealed. Controlled disruption is therefore diagnostically useful when it identifies which pre-specified function failed, which cue or mapping was involved, and whether reconfiguration improves transfer. Error tolerance by itself is not the target.

Simulation is one way to create such probes. Waddell et al. (2025) show how acoustic, visual, and social features can be combined in a performance simulator. For MAR, the important design question is whether the simulation varies a performance-relevant relation and is followed by transfer outside the trained setup. A more elaborate simulator is not automatically a better perturbation.

For advanced performers, practice planning can include a bounded robustness phase after acquisition, interpretation, and memory are sufficiently stable. Its value may lie less in occupying large amounts of practice time than in identifying the small number of conditions under which the performance is fragile, then testing whether targeted recalibration reduces that fragility.

The framework may also offer one testable interpretation of why two performers who look equally prepared in a studio respond differently in an unfamiliar venue or evaluative setting. Temperament, anxiety, and accumulated experience remain plausible explanations. MAR adds a process question: do the performers differ in detection, available solutions, attention, replanning, regulation, or cue redundancy when the condition changes?

## 10 Discussion

MAR organizes a transfer problem that existing expertise, motor-learning, and self-regulation accounts already partly explain. Its value lies in linking prospectively demonstrated options and process-specific responses to independently scored musical outcomes under changed conditions. It remains a proposed synthesis whose incremental explanatory value requires comparison with established measures.

The priority is to test the diagnostic claims in Propositions 2, 3, and 8 using repeated baselines, reliable implementation measures, independent classification of representativeness, and pre-mapped cues. Studies should compare MAR predictors with corresponding measures from neighboring accounts, not only with baseline skill. Repeated null or reversed findings under adequate designs would leave the framework descriptive rather than establish a distinct explanation.

The reviewed piano, simulation, and attention studies provide starting points for those tests, not validation of a unitary reserve (Shao et al., 2026; Waddell et al., 2025; Bae, 2026; Hohagen and Immerz, 2024). Evidence across instruments, voice, genres, and interactive settings remains necessary to assess scope.

RPP also inherits the learning constraints reviewed in Section 3: acquisition fluency can mislead judgments of transfer, task-irrelevant variability can impair learning, and challenge must be matched to skill (Schmidt and Bjork, 1992; Ranganathan and Newell, 2013; Dhawale et al., 2017; Cardis et al., 2018; Guadagnoli and Lee, 2004). Representativeness is a design hypothesis to test, not a guarantee of benefit (Pinder et al., 2011).

The prospective endpoint deliberately limits the framework. Preservational adaptation tests continuity of a declared function, while transformative adaptation may produce a valuable new conception. Prospective meta-criteria can bring the latter within an extended MAR design; unanticipated transformations remain separate exploratory outcomes. This preserves falsifiability without reducing artistic value to fidelity to an initial intention.

The five-capacity decomposition is likewise provisional. Evidence of overlapping indicators, weak discriminant validity, or omitted processes should change the model rather than be absorbed into an unspecified global reserve. Perturbation-specific profiles and coupling estimates offer a way to test both the proposed boundaries and their practical value.

All applications remain bounded by performance health. Recognizing unsafe demands, modifying the task, seeking support, or stopping can be appropriate responses; maintaining output at a physical or psychological cost is not successful adaptation.

## 11 Conclusion

Dependable musical performance requires a stable conception and options for responding when familiar conditions change. MAR organizes those options as five provisional, partially independent capacities supported by structural anchors, with their coupling determined by the task and perturbation.

The proposed framework should be judged by whether its prospective operationalization improves explanation of perturbation cost, recalibration, and transfer beyond established predictors. Its diagnostic claims do not imply a unique faculty or a single latent reserve.

RPP translates the framework into bounded transfer probes after basic stability: specify a criterion, introduce a representative change, diagnose, reconfigure, re-test, and transfer. Preservation and artistic transformation remain distinguishable outcomes. Practicing for the unexpected means preparing conditional responses while testing what they actually preserve.
